# Supplementary material for: Increased H. pylori stool shedding and EPIYA-D cagA alleles are associated with gastric cancer in an East Asian hospital
Source: PLoS One. 2018 Sep 12;13(9):e0202925. doi: 10.1371/journal.pone.0202925 (PMC6135355; doi:10.1371/journal.pone.0202925)
Supplement: S1 Questionnaire — (DOCX) [file pone.0202925.s001.docx]

**H.P项目信息采集表** 日期: _____________

非常感谢您的合作，请您回答以下几个关于您的健康和基本信息的问题。请您尽可能准确的回答；这将对我们的工作非常有帮助。如果您不愿意回答某一问题，请选择拒绝回答项。

**A.基础信息**

**A1.** 研究对象编号: ____________________________

**A2.** 年龄: ________ 拒绝回答

**A3.** 性别: 女 男 拒绝回答

**A4.** 民族：_____________ 拒绝回答

**A5.** 您出生在哪个省市？ __________________________ 拒绝回答

**A6.** 您现在的居住地? ___________________ 拒绝回答

**A7.** 您在当前居住地居住了多久？ ___________________ 拒绝回答

**B.健康状况及药物治疗**

**B1-1.** 你有没有因为幽门螺杆菌感染而被治疗？

有 →

没有

不知道

拒绝回答

**B1-2.** 如果有，请注明治疗时间 _____________

**B2.** 您是否被诊断过有以下病症？

胃酸/反胃  胃和十二指肠溃疡

食道炎 胃癌

食道癌  无 拒绝回答

**B3.** 你经常口服抗生素吗？

长期服用（每天服用）

每月服用

每年7到11次

每年2到六次

每年一次

很少服用

拒绝回答

**B4.**你现在服用任何药物吗？

否

是

拒绝回答

如果是，请在以下空白处列出

**B4-1** 药物 1 ________________________

**B4-2**药物 2 ________________________

**B4-3**药物 3 ________________________

**B4-4**药物 4 ________________________

**B4-5**药物 5 ________________________

**签名**：_____________
