# Supplementary material for: Increased H. pylori stool shedding and EPIYA-D cagA alleles are associated with gastric cancer in an East Asian hospital
Source: PLoS One. 2018 Sep 12;13(9):e0202925. doi: 10.1371/journal.pone.0202925 (PMC6135355; doi:10.1371/journal.pone.0202925)
Supplement: S2 Questionnaire — (DOCX) [file pone.0202925.s002.docx]

**Evaluation of a New, Non-invasive *H. pylori* Genotyping Method**

Thank you for taking the time to complete this health and demographics questionnaire. Please answer all of the questions as best you can. You do not have to answer any question you do not wish to answer, but if you could indicate “prefer not” to any questions you wish not to answer, this would be helpful to us.

Survey date: _____________

**A1:** Study ID Number: ____________________

**Demographic Questions**

**A2:** Age: ________ Prefer not to answer

**A3:** Gender: Female Male Prefer not to answer

**A4:** Which of the following best describes your ethnic group? Please mark one or more.

Han

Hui

Other, please specify ___________________________

Not sure

Prefer not to answer

**A5:** In what region (province, municipality, or special administrative region) were you born? __________________________

Prefer not to answer

**A6:** Which city do you live now ___________________

Prefer not to answer

**A7:** how long have you lived in here?

Less than one month

1 to 6 months

7 to 12 months

More than 1 year up to 2 years

More than 2 years up to 5 years

More than 5 years up to 10 years

More than 10 years

Other

**Medical Conditions and Medications**

**B1-1:** Have you ever been treated for *Helicobacter pylori* infection?

Yes →

No

Not sure

Prefer not to answer

**B1-2:** if yes, when were you treated? _____________

**B2:** Have you ever been diagnosed with any of the following conditions?

Please mark all that apply.

Heartburn/reflux

Ulcers in the stomach or small intestine (duodenum)

Esophagitis

Gastric cancer

Esophageal cancer

None

Prefer not to answer

**B3:** How often do you take a course of oral antibiotics (pills)?

On long-term course (taken daily)

Monthly

7 to 11 times per year

2 to 6 times per year

Yearly

Less than yearly

Prefer not to answer

**B4:** Are you currently taking any medications?

Yes →

No

Prefer not to answer

Please list

**B4-1:** Medication 1 ________________________

**B4-2:** Medication 2 ________________________

**B4-3:** Medication 3 ________________________

**B4-4:** Medication 4 ________________________

**B4-5:** Medication 5 ________________________
